# Early hippocampal hyperexcitability precedes synaptic reorganization in mouse models of amyloidosis

**DOI:** 10.1002/alz.092939

**Published:** 2025-01-03

**Authors:** Ajit Ray, Iulia Loghinov, Alison L Barth

**Affiliations:** ^1^ Carnegie Mellon University, Pittsburgh, PA USA

## Abstract

**Background:**

The failure of amyloid plaque‐reducing drugs to reverse cognitive decline in Alzheimer’s disease (AD) has suggested that treatments might be more effective in early or prodromal stages of the disease. However, the progression of synaptic and circuit changes associated with Aβ overexpression, particularly at very early ages, have not been well‐characterized. Indeed, evidence from both human and animal studies indicates that brain structure and function might be altered months to years before plaques can be detected.

**Method:**

Here we used quantitative, fluorescence‐based methods for synapse detection in CA1 pyramidal neurons (Pyr) to investigate the interaction between abnormal circuit activity, measured by Fos‐immunoreactivity (Fos‐IR), and synapse reorganization in mouse models of amyloidosis.

**Result:**

Using a fluorescently‐tagged molecule (FAPpost) for both excitatory and inhibitory synapses, we find that Aβ overproduction is associated with early synapse gain and loss, depending on the dendritic compartment. Juvenile APP/PS1 and Tg2576 transgenic mice both show a reduction in synapses at the apical tuft of CA1 Pyr and an increase in synapse density along the apical dendrite, which receives inputs from CA3 in animals. These effects were observed at just 6 weeks of age, well before cognitive deficits and amyloid plaques can be detected. Elevated hippocampal activity precedes this reorganization, with elevated Fos‐IR in both CA3 and CA1 present at weaning.

**Conclusion:**

These data indicate that elevated Aβ may initiate abnormal activity and subsequently input‐specific synapse plasticity. Taken together, our findings indicate that sustained amyloidosis drives heterogeneous and progressive circuit‐wide abnormalities.

